# Ionoacoustic tomography of the proton Bragg peak in combination with ultrasound and optoacoustic imaging

**DOI:** 10.1038/srep29305

**Published:** 2016-07-07

**Authors:** Stephan Kellnberger, Walter Assmann, Sebastian Lehrack, Sabine Reinhardt, Peter Thirolf, Daniel Queirós, George Sergiadis, Günther Dollinger, Katia Parodi, Vasilis Ntziachristos

**Affiliations:** 1 Institute for Biological and Medical Imaging, Technische Universität München and Helmholtz Zentrum München, 85764 Neuherberg, Germany; 2 Cardiovascular Research Center, Cardiology Division, Massachusetts General Hospital, Harvard Medical School, Boston, Massachusetts 02114, USA; 3 Department for Medical Physics, Ludwig-Maximilians-Universität München, 85748 Garching, Germany; 4 Department of Electrical and Computer Engineering, Aristotle University, 54124 Thessaloniki, Greece; 5 Institute for Applied Physics and Measurement Technology, Universität der Bundeswehr, 85577 Neubiberg, Germany

## Abstract

Ions provide a more advantageous dose distribution than photons for external beam radiotherapy, due to their so-called inverse depth dose deposition and, in particular a characteristic dose maximum at their end-of-range (Bragg peak). The favorable physical interaction properties enable selective treatment of tumors while sparing surrounding healthy tissue, but optimal clinical use requires accurate monitoring of Bragg peak positioning inside tissue. We introduce ionoacoustic tomography based on detection of ion induced ultrasound waves as a technique to provide feedback on the ion beam profile. We demonstrate for 20 MeV protons that ion range imaging is possible with submillimeter accuracy and can be combined with clinical ultrasound and optoacoustic tomography of similar precision. Our results indicate a simple and direct possibility to correlate, *in-vivo* and in real-time, the conventional ultrasound echo of the tumor region with ionoacoustic tomography. Combined with optoacoustic tomography it offers a well suited pre-clinical imaging system.

Range uncertainties are among the greatest issues in ion beam therapy, and can have very different reasons: on the one hand range calculation inaccuracies in treatment planning (e.g. stopping power conversion from X-ray Computed Tomography [CT] images, CT artifacts), on the other hand inaccuracies of the actual irradiation (e.g. positioning of the patient and organ motion, physiological changes)^1^,^2^,^3^. To compensate for the uncertainty in precisely placing the proton beam within tissue, safety margins are set around the tumor volume in treatment planning (typically 3.5% of the proton beam range^1^), limiting the overall ability of proton therapy to provide maximum dose to the tumor and sparing surrounding healthy tissue^4^.

The importance of feedback during ion beam delivery is underscored by the consideration of various techniques for non-invasive localization of the Bragg peak position, typically based on the detection of nuclear reactions accompanying proton irradiation of tissue. Positron emission tomography (PET) is perhaps the most advanced method currently under clinical investigation^5^,^6^, whereas the detection and imaging of prompt reaction gammas is just entering clinical testing^7^,^8^. Both methods rely, however, on complex and bulky detector instrumentation and deliver only indirect information about the Bragg peak position. Another field, the development of compact laser-driven accelerators for ion-beam therapy^9^,^10^, could similarly benefit by a technique that gives real-time feedback to the beam profile and energy deposition characteristics in order to optimize, calibrate, and standardize ion beam technology.

In this work we introduce ionoacoustic tomography (IAT) as a potent modality for high-resolution characterization of the Bragg peak spatial distribution^11^. We hypothesized that by detecting and mathematically inverting the ultrasound waves produced upon transient stopping of ions by water/tissue we could reconstruct the deposited dose profile. Such approach could yield a direct measure of energy deposition and enable ultrasound-diffraction limited imaging resolution (100–300 micrometers) within several centimeters penetration depth. The induction of acoustic waves using proton beams was first reported by Sulak *et al.*^12^ in 1979, i.e. in the same time frame where optoacoustic (photoacoustic) imaging emerged^13^. Follow–up studies confirmed ultrasound detection from soft tissues even during tumor treatment, but were limited to single element acquisition and very low signal strength^14^. Using high frequency (up to 10 MHz) ultrasound transducers, we have recently shown one dimensional detection of ionoacoustic signals from a 20 MeV proton Bragg peak^15^. Besides several ionoacoustic simulation studies^16^,^17^,^18^, in experiments at an advanced hospital-based cyclotron with 230 MeV protons ionoacoustic signals were observed around 10 kHz with hydrophones^19^. Alsanea *et al.*^20^ demonstrated ionoacoustic tomography in simulations, however, no experimental study so far has revealed the spatial characteristics of proton beams. Here, we demonstrate submillimeter resolution three dimensional (3D) ionoacoustic tomography of the Bragg peak. We further combine IAT with ultrasound and optoacoustic imaging^21^,^22^,^23^,^24^ to demonstrate that not only Bragg peak profiles can be resolved with high resolution, but that they can also be co-registered to the underlying tissue morphology.

## Results

### Ionoacoustic imaging setup

To experimentally prove IAT we employed proton beams of 20 and 21 MeV provided by the Tandem accelerator of the Maier-Leibnitz-Laboratory (see Methods). According to Geant4 simulations^25^, protons at this energy deliver a spatially confined Bragg peak with full-width-half-maximum (FWHM) of about 0.3 mm in beam direction and a range of approximately 4 mm in water, ideal for ionoacoustic characterization studies. Ionoacoustic signals were induced using a beam focus of about 1 mm FWHM (Supplementary Fig. S3(a)) and a proton pulse length of less than 200 ns, satisfying both thermal and stress confinement conditions (see Methods). For 2D raster scanning, we based image reconstruction of the Bragg peak on a maximum intensity projection of the acquired ionoacoustic data set while for 3D tomographic imaging we used the modified backprojection algorithm^26^ or the model based reconstruction^27^, similar to inversion methods applied in optoacoustic tomography^28^,^29^.

### 2D ionoacoustic imaging of the Bragg peak

**Figure 1:**
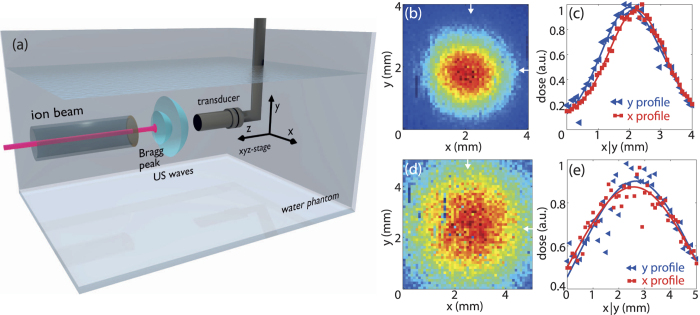
Experimental setup for 2D ionoacoustic imaging of proton beams. (**a**) Schematic of the 2D raster scan system using a focused high resolution acoustic wave sensor to characterize the proton dose distribution in water. Increased energy losses could be induced by means of an aluminum absorber inserted into the beam path. (**b**) Maximum intensity projection after raster scanning the proton beam. Arrows mark the position of the maximum. (**c**) Line profile of the Bragg peak in *x* and *y* direction, showing the measurement points (triangle and squares) and Gaussian fits to calculate the full width half maximum (FWHM). In *x*-direction, we determined the FWHM to be 2.4 ± 0.3 mm (red line) and in *y*-direction 2.3 ± 0.3 mm (blue line). (**d**) Bragg beak characterization with Al absorber. Maximum intensity projection of the particle beam after introducing a 0.5 mm Al sheet in the beam axis immediately after the vacuum exit window, arrows mark the maximum of the Bragg peak. (**e**) Line profile of the Bragg peak in *x* and *y* direction, illustrating scanning points and Gaussian fits to determine the FWHM.

We first investigated the capability to image the Bragg peak with IAT in a two dimensional (2D) setting. Figure 1(a) shows the experimental 2D setup based on an uncalibrated 10 MHz single element focused ultrasound transducer located at focal distance of 25 mm from the expected Bragg peak position. We scanned the Bragg peak over a 4 × 4 mm^2^ field of view in step sizes of 80 μm. Figure 1(b) shows the maximum intensity projection (MIP), revealing a FWHM along the *x*-*y* axis of 2.4 ± 0.3 mm and 2.3 ± 0.3 mm as illustrated in Fig. 1(c). These values are in good agreement with a Geant4 simulation based on the beam size measured with a radiochromic film located at the water phantom entrance foil (Supplementary Fig. S2(a)). Using k-Wave^30^ to simulate ionoacoustic signal generation and propagation we found that 10^6^ particles per pulse, corresponding to 1 μJ stored energy in the Bragg peak volume generated a pressure wave of 67 Pa at the detector position. The induced ionoacoustic signals exhibit a signal to noise ratio (SNR) of about 40 dB after averaging 16 signals. These numbers are similar to SNR values measured in Assmann *et al.*^15^.

Upon confirming the fundamental ability to directly image the Bragg peak position and proton absorption distribution, we investigated the IAT detection sensitivity by introducing a 0.5 mm thick aluminum absorber in the proton beam after the vacuum exit before the 8 cm air gap. Geant4 simulations predict about a factor of two increase of the lateral spread due to the introduction of absorbing material into the beam (Supplementary Fig. S2(b)). Using IAT, we confirmed this prediction (Fig. 1(d,e)), revealing a beam profile measuring FWHM *x* = 5.4 ± 0.5 mm and *y* = 5.3 ± 0.5 mm. Due to the lower energy density in this experiment, SNR decreased to approximately 30 dB after averaging 16 events.

### 3D ionoacoustic tomography

**Figure 2:**
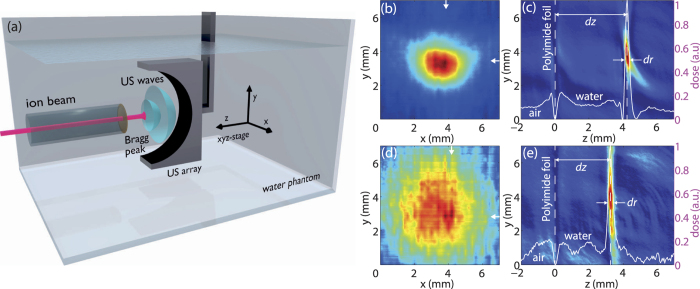
Three-dimensional tomographic scan of the Bragg peak. (**a**) Experimental setup for 3D ionoacoustic imaging. (**b**) Maximum intensity projection of the 3D reconstruction in the *xy* plane. The arrows indicate the position of the maximum in *x* and *y* axis. (**c**) Image of the reconstructed volume, showing the MIP in the *yz*-plane. The line profile depicts the position of the Bragg peak at a distance of 4.3 ± 0.2 mm from the polyimide foil and further reveals a longitudinal FWHM of the Bragg peak of 0.28 ± 0.05 mm. (**d**) Image of the Bragg peak after introducing an aluminum absorber in the beam path. The arrows indicate the position of the Bragg peak maximum. (**e**) Proton range determination using ionoacoustic tomography. The inserted aluminum absorber reduces the range of protons in water.

To investigate whether Bragg-peak images could be captured in three dimensions and in video-rate mode, we employed (Fig. 2(a)) an ultrasonic 64 element curved array (172° half circle, center frequency 5 MHz) typically applied in optoacoustic tomography^28^,^31^. The ultrasound array was moved in lateral steps of 200 μm over the particle beam, acquiring a 3D data set of generated ultrasonic waves. Image reconstruction yielded a 301 × 301 × 36 volume element (voxel) image with a grid spacing of 50 μm which was used to visualize the stopped particles (see Methods). These first three dimensional reconstructions of the Bragg peak revealed the entire spatial distribution of particle beams in water (Supplementary Movie S1). The MIP along the beam axis, illustrated in Fig. 2(b) (and Supplementary Fig. S1(a)), reveals a FWHM in *x* and *y*-direction of 2.8 ± 0.3 mm and 2.0 ± 0.3 mm, in accordance with the related radiochromic film measurement (Supplementary Fig. S3(c,d)). The MIP in *yz* plane, shown in Fig. 2(c), visualizes the range of protons in water with the Bragg peak position at *dz* = 4.3 ± 0.2 mm and the FWHM of the Bragg peak of *dr* = 0.28 ± 0.05 mm. This proton range is in full agreement with earlier 1D measurements^15^ and Geant4 simulation results (Supplementary Fig. S2(a)). The effects of introducing a 0.5 mm thick aluminum absorber could be monitored in three dimensions (Fig. 2(d,e); Supplementary Movie S2). The increased lateral spread of stopped particles (FWHM of 6.2 ± 0.5 mm and 6.1 ± 0.5 mm in *x* and *y* axis) was confirmed by the corresponding simulations (Supplementary Fig. S2(b)) and was accompanied by a reduced Bragg peak range of *dz* = 3.3 ± 0.2 mm, and a FWHM of the Bragg peak along the beam axis of *dr* = 0.35 ± 0.05 mm.

### Multimodal ionoacoustic, optoacoustic, and ultrasound imaging

**Figure 3:**
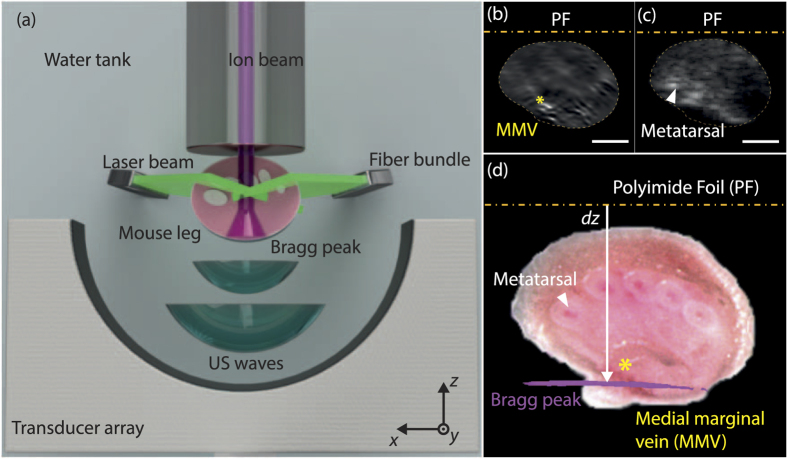
Triple-modality imaging of a mouse leg using optoacoustics, ionoacoustics, and ultrasonography. (**a**) Schematic of the opto- and ionoacoustic experiment. For ultrasonography we replaced the curved array with a linear US-array (picture not shown). (**b**) Optoacoustic reconstruction of a mouse leg positioned in the proton beam line (scale bar represents 2 mm, star marks the medial marginal vein). (**c**) Ultrasonography of the mouse leg, showing metatarsal bones (scale bar represents 2 mm). (**d**) Cryoslice of a mouse leg with the ionoacoustic reconstruction (magenta color) co-registered to the optical image, displaying the Bragg peak at the distal end of the leg with a proton range *dz* = 4.7 ± 0.2 mm (star marks the medial marginal vein).

The ability to image the Bragg peak in three dimensions comes with significant implications in terms of feedback during treatment. For accurate IAT application it would be therefore important to register the ion-beam profile onto the underlying tissue morphology. For this reason we combined IAT with ultrasound and optoacoustic imaging. Initial experiments were performed using 21 MeV proton energy for IAT and 532 nm laser illumination on an immobilized mouse leg *ex-vivo*, under identical placement conditions (Fig. 3(a)). By exchanging the IAT transducer for a co-localized linear ultrasound (US) array we also acquired pulse echo images from the same leg location. Optoacoustic images (Fig. 3(b)) revealed optical absorption morphology showing the medial marginal vein. Ultrasound imaging, depicted in Fig. 3(c), further revealed anatomical features, such as bone structures that can be co-registered with IAT and optoacoustic images. An invasive cryo-slice image of the mouse leg, shown in Fig. 3(d), demonstrated good agreement with the optoacoustic and ultrasound image. Furthermore, Fig. 3(d) shows the co-registration of the IAT image onto the optical image, illustrating the Bragg peak position reaching the distal end of the mouse leg with a proton range of *dz* = 4.7 ± 0.2 mm in the mouse leg tissue. We note that an increased lateral spreading of the Bragg peak as shown in Fig. 3(d) can be attributed to lower SNR compared to previous experiments due to soft tissue effects. The longer range of protons in tissue compared to water experiments (Geant4 simulation predicts *dz* = 4.5 mm at 21 MeV proton energy) can be explained by the slightly different properties of the leg tissue over water. The registration of IAT signals on other tissue images may pave the way towards monitoring of beam quality and proton therapy treatments^32^.

### Discussion

Our results effectively establish ionoacoustic tomography as a potent tool that could be utilized in multiple applications within the proton-beam development and proton-therapy fields. We present a novel experimental arrangement to interrogate two abilities. The first feature is related to the so far unknown capacity to generate images of proton beams stopped within tissues and tissue-like media. Such capacity could be fundamental to enable on-the-fly optimization of beam delivery during proton treatment. Moreover, IAT could be uniquely suited for characterization of the very intense beam pulses of future compact laser-driven accelerators^9^,^10^,^33^ due to its large dynamic range and the temporal separation of the acoustic signal from the laser pulse induced electromagnetic pulse (EMP). The second ability is related to the development of a hybrid system that would allow the unique co-localization of a proton beam and the underlying tissue anatomy. Such system could be employed during treatment regimens to register experimentally measured proton beams onto the underlying tissue morphology so as to optimize dose delivery. The acoustic waves generated in response to the sharp distal falloff of stopped protons are spatiotemporally analyzed to produce two-dimensional and three-dimensional maps and videos of proton range and Bragg peak shape, resulting in submillimeter accuracy. In particle therapy, the combination of ultrasonography and IAT can enable accurate positioning of the ion beam on tissue morphology only or jointly with ultrasonic markers. As both methods are based on ultrasound effects, inhomogeneities occurring in soft tissue can be neglected in IAT. Moreover, optoacoustic tomography may be a valuable imaging combination for pre-clinical particle therapy research well adapted to small animal morphology thanks to its high spatial resolution or for clinical imaging of superficial diseases, in particular for depths of up to 3 cm (e.g breast cancer visualization)^34^,^35^.

## Methods

### Proton beam parameters and radiochromic films

We used proton energies of 20 and 21 MeV with an energy resolution better than 0.1% delivered by the 12 MV electrostatic Tandem accelerator of the Maier-Leibnitz-Laboratory (LMU and TU München). To enable high image quality, we set the beam current to about 3 nA measured with a Faraday cup at the beam vacuum exit. This current corresponds to 10^6^ particles per pulse being equivalent to 1 μJ per proton pulse, one order of magnitude above the detection limit of 10^5^ particles per pulse as previously determined by Assmann *et al.*^15^ in a similar ionoacoustic setup. For our 2D scan using 16 averages, this beam current resulted in a total energy of approximately 66 mJ while for the 3D scan averaging 512 events, a total energy of about 1.2 mJ was used.

The beam focus size was adjusted to be about 2 mm^2^ at the entrance foil of the water phantom. The beam focus could be seen on a cesium iodide (CsI) scintillating screen during beam tuning and was measured after specific irradiations with a radiochromic film (GafChrom). Radiochromic films offer a simple and independent possibility to check the beam spot size immediately after entrance into the water phantom. A scan of the irradiated area (Fig. S3(a,c)) allows one to determine the intensity distribution in *x* and *y* (Fig. S3(b,d)) which can be directly compared to the corresponding values of ionoacoustic scans (Fig. 1(b,d)). At our beam intensity, a film exposure took about 30 sec followed by scanning with a photo scanner (Epson Perfection V700) using the full physical scan resolution of 1200 dpi^36^. Measured dose profiles corresponding to Figs 1(b) and 2(b) are presented in Supplementary Information, Fig. S3 where different lateral resolutions (radiochromic film ~50 μm, ultrasound transducer ~0.3 mm) and acquisition times (film ~30 sec, transducer scan duration ~1.5 h suffering from long term beam instabilities) of the methods have to be taken into account.

### IAT setup

All IAT measurements were performed by directing the proton beam from the Tandem accelerator through a vacuum exit window and an 8 cm air gap into a water tank (33 × 17 × 19 cm^3^) through a 50 μm polyimide window. The water tank contained different ultrasound sensors employed to study IAT feasibility and performance of proton beam characterization at different dimensions or for performing co-registered ultrasound and optoacoustic imaging.

Two dimensional images of the particle beam profile were enabled by raster scanning a spherically focused ultrasound sensor (V311, focal distance 25.4 mm, central frequency 10 MHz, bandwidth 100%, Olympus-Panametrics, USA) mounted on a *xyz* stage with 10 μm accuracy. The 10 MHz transducer had a beam width of 293 μm with a length of the focal zone of 4 mm at its central frequency. Raster scans were performed using a scanning distance of *dx* = *dy* = 80 μm for the Bragg peak characterization illustrated in Fig. 1(b) and *dx* = *dy* = 100 μm for the aluminum experiment shown in Fig. 1(d). A scan with 250 raster points took around 1.5h, during this time the beam intensity as well as the focus size fluctuated by ± 30%, therefore an increase of the measured focus size has to be expected. At its central frequency, the detector has a −6 dB beam diameter at focal length of about 0.3 mm, which limits the lateral resolution additionally. The water temperature was kept around 35 °C and continuously monitored for US velocity calculation. We amplified ionoacoustic signals employing a low noise 63 dB amplifier (AU 1291, Miteq Inc., USA) and additionally increased SNR in Fig. 1(b,d) by averaging 16 waveforms. Signals were acquired using a digital oscilloscope set to a sampling rate of 500 MS/s.

Fast 2D and 3D imaging of the particle beam were performed using a 64 element curved US array (172° half circle, central frequency 5 MHz, bandwidth > 50%, Vermon, France) employed in previous optoacoustic imaging studies^22^,^28^,^37^. The in-plane spatial resolution of the US-array is estimated to be in the order of 150 μm while elevational resolution is approximately 800 μm. To increase SNR, we used a homebuilt amplifier array consisting of 64 elements offering a gain of 52 dB. Additionally, signals were averaged 512 times for each scanning step, resulting in a frame rate of more than 1 frame per second.

Ultrasonography was performed employing a commercially available 128-element linear array (7.5 MHz, 12L5V linear array, Model Terason t2000, Terason, Burlington, MA, USA) which replaced the curved US array used for optoacoustic and ionoacoustic imaging. We note that the US images were not perfectly co-registered to the optoacoustic images and invasive cryo-slice images of the mouse leg after exchanging the iono/optoacoustic imaging array with the US array. This mismatch is attributed to the spatially limited geometry of our experimental imaging system, but can be improved in future experiments.

### Image reconstruction

We used different reconstruction algorithms to generate ionoacoustic images. Maximum intensity projections of the two dimensional raster scans illustrated in Fig. 1(b,d) were generated by determining the peak-to-peak difference of the ionoacoustic signal at the Bragg peak position. Three dimensional representations of the Bragg peak were reconstructed using the modified backprojection algorithm^26^ for Fig. 3(b) or the model based reconstruction^27^ for Fig. 2.

### *Ex-vivo* mouse imaging

All mouse procedures were approved by the Bavarian Animal Care and Use Committee and all experiments were performed in accordance with the guidelines and regulations approved by the Government of Bavaria, Germany. Iono- and optoacoustic imaging was performed on a CD-1 nude mouse sacrificed prior to the experiment. We imaged the mouse leg taking into account the limited range of protons at 21 MeV which relates to *dz* = 4.5 mm in water. The mouse was mounted on a custom built animal holder and illuminated from one side using three fiber bundles connected to a 532 nm laser. The measurement protocol comprised optoacoustic imaging of the mouse leg followed by ionoacoustic imaging to obtain the position of the Bragg peak. Finally, optoacoustic imaging of the mouse leg at a similar position was performed.

### Thermal and stress confinement conditions

According to Wang^38^, the characteristic dimension of the heated region is the axial FWHM of the Bragg peak with about 300 μm. The related thermal relaxation time amounts to 570 ms, whereas the thermal stress relaxation time (using an US velocity of 1500 m/s) is 200 ns. Therefore, thermal and stress confinement conditions are fulfilled with proton pulse lengths below 200 ns at the considered energies.

### Geant4 simulation

For calculating the proton dose deposition in water, we used Geant4^25^ (version 10.0.p01) and activated the QGSP_BIC_HP 2.0 physics lists for handling of the main electromagnetic and nuclear processes. A grid size between 10 μm and 100 μm was chosen for all three scoring dimensions. The value of the ionization potential of water was set to 78 eV, according to the latest recommendation of Andreo *et al.*^39^. Due to the low proton beam energies used in our ionoacoustic experiments, inaccuracies related to the density and the ionization potential of water could be neglected in our Bragg peak position simulations^15^. We found that uncertainties of the simulation can mainly be attributed to inaccurate determination of the experimental settings in the beam path, such as the thickness of the polyimide foil or the air gap, resulting in ~30 μm total error. Examples of Geant4 simulations for 20 MeV protons in water are shown in Supplementary Information, Fig. S2 (axial dose distribution, straggling without/with Al absorber).

### k-Wave simulation

The k-Wave simulation engine was used to simulate the entire ionoacoustic propagation and detection model, including proton energy transfer to the medium, generation of acoustic waves, and the propagation of acoustic waves until detection^27^. For this purpose, we supplied the k-Wave toolbox with the output of our Geant4 simulation. Correspondingly, the forward model comprised a 3D matrix with 30 × 30 × 10 μm^3^ grid size spacing (similar to the used Geant4 scoring), and a volume size of 13.2 × 13.2 × 32 mm^3^ in corresponding *x*, *y*, and *z* direction.

## Additional Information

**How to cite this article**: Kellnberger, S. *et al.* Ionoacoustic tomography of the proton Bragg peak in combination with ultrasound and optoacoustic imaging. *Sci. Rep.* **6**, 29305; doi: 10.1038/srep29305 (2016).

## Supplementary Material

Supplementary Movie S1

Supplementary Movie S2

Supplementary Information

## References

[b1] Paganetti H. Range uncertainties in proton therapy and the role of Monte Carlo simulations. Phys Med Biol 57, R99–R117 (2012). 22571913 10.1088/0031-9155/57/11/R99 PMC3374500

[b2] Knopf A.-C. & Lomax A. *In vivo* proton range verification: a review. Phys Med Biol 58 (2013). 10.1088/0031-9155/58/15/R131 23863203

[b3] Schuemann J., Dowdell S., Grassberger C., Min C. H. & Paganetti H. Site-specific range uncertainties caused by dose calculation algorithms for proton therapy. Phys Med Biol 59, 4007–4031 (2014). 24990623 10.1088/0031-9155/59/15/4007 PMC4136435

[b4] Wilson R. R. Radiological use of fast protons. Radiology 47, 487–491 (1946). 20274616 10.1148/47.5.487

[b5] Parodi K. *et al.* Patient study of *in vivo* verification of beam delivery and range, using positron emission tomography and computed tomography imaging after proton therapy. International Journal of Radiation Oncology Biology Physics 68, 920–934 (2007). 10.1016/j.ijrobp.2007.01.063 PMC2047826 17544003

[b6] Parodi K. Vision 20/20: Positron emission tomography in radiation therapy planning, delivery, and monitoring. Medical physics 42, 7153, doi: 10.1118/1.4935869 (2015). 26632070

[b7] Min C.-H., Kim C. H., Youn M.-Y. & Kim J.-W. Prompt gamma measurements for locating the dose falloff region in the proton therapy. Appl Phys Lett 89 (2006).

[b8] Hueso-Gonzalez F. *et al.* First test of the prompt gamma ray timing method with heterogeneous targets at a clinical proton therapy facility. Phys Med Biol 60, 6247–6272, doi: 10.1088/0031-9155/60/16/6247 (2015). 26237433

[b9] Malka V. *et al.* Principles and applications of compact laser-plasma accelerators. Nat Phys 4, 447–453 (2008).

[b10] Hooker S. M. Developments in laser-driven plasma accelerators. Nat Photonics 7, 775–782 (2013).

[b11] Parodi K. & Assmann W. Ionoacoustics: A new direct method for range verification. Modern Physics Letters A 30, doi: 10.1142/s0217732315400258 (2015).

[b12] Sulak L. *et al.* Experimental studies of the acoustic signature of proton-beams traversing fluid media. Nuclear Instruments & Methods 161, 203–217 (1979).

[b13] Rosencwaig A. Photoacoustic spectroscopy of biological materials. Science 181, 657–658 (1973). 4353357 10.1126/science.181.4100.657

[b14] Hayakawa Y. *et al.* Acoustic pulse generated in a patient during treatment by pulsed proton radiation beam. Radiation Oncology Investigations 3, 42–45 (1995).

[b15] Assmann W. *et al.* Ionoacoustic characterization of the proton Bragg peak with submillimeter accuracy. Medical physics 42, 567–574 (2015). 25652477 10.1118/1.4905047

[b16] Terunuma T. *et al.* Waveform simulation based on 3D dose distribution for acoustic wave generated by proton beam irradiation. Medical physics 34, 3642–3648 (2007). 17926968 10.1118/1.2767985

[b17] Jones K. C., Witztum A., Sehgal C. M. & Avery S. Proton beam characterization by proton-induced acoustic emission: simulation studies. Phys Med Biol 59, 6549–6563, doi: 10.1088/0031-9155/59/21/6549 (2014). 25322212

[b18] Jones K. C., Seghal C. M. & Avery S. How proton pulse characteristics influence protoacoustic determination of proton-beam range: simulation studies. Phys Med Biol 61, 2213–2242, doi: 10.1088/0031-9155/61/6/2213 (2016). 26913839

[b19] Jones K. C. *et al.* Experimental observation of acoustic emissions generated by a pulsed proton beam from a hospital-based clinical cyclotron. Medical physics 42, 7090–7097, doi: 10.1118/1.4935865 (2015). 26632062

[b20] Alsanea F., Moskvin V. & Stantz K. M. Feasibility of RACT for 3D dose measurement and range verification in a water phantom. Medical physics 42, 937–946, doi: 10.1118/1.4906241 (2015). 25652506

[b21] Razansky D. *et al.* Multispectral opto-acoustic tomography of deep-seated fluorescent proteins *in vivo*. Nat Photonics 3, 412–417 (2009).

[b22] Razansky D., Buehler A. & Ntziachristos V. Volumetric real-time multispectral optoacoustic tomography of biomarkers. Nat Protoc 6, 1121–1129 (2011). 21738125 10.1038/nprot.2011.351

[b23] Wang L. V. & Hu S. Photoacoustic tomography: *in vivo* imaging from organelles to organs. Science 335, 1458–1462 (2012). 22442475 10.1126/science.1216210 PMC3322413

[b24] Wang L. V. Multiscale photoacoustic microscopy and computed tomography. Nat Photonics 3, 503–509 (2009). 20161535 10.1038/nphoton.2009.157 PMC2802217

[b25] Agostinelli S. *et al.* GEANT4-a simulation toolkit. Nuclear Instruments & Methods in Physics Research Section a-Accelerators Spectrometers Detectors and Associated Equipment 506, 250–303 (2003).

[b26] Xu M. H. & Wang L. V. Universal back-projection algorithm for photoacoustic computed tomography (vol 71, art no 016706, 2005). Phys Rev E 75 (2007). 10.1103/PhysRevE.71.016706 15697763

[b27] Rosenthal A., Razansky D. & Ntziachristos V. Fast semi-analytical model-based acoustic inversion for quantitative optoacoustic tomography. IEEE Trans Med Imaging 29, 1275–1285 (2010). 20304725 10.1109/TMI.2010.2044584

[b28] Buehler A., Herzog E., Razansky D. & Ntziachristos V. Video rate optoacoustic tomography of mouse kidney perfusion. Optics letters 35, 2475–2477 (2010). 20634868 10.1364/OL.35.002475

[b29] Lutzweiler C. & Razansky D. Optoacoustic imaging and tomography: reconstruction approaches and outstanding challenges in image performance and quantification. Sensors 13, 7345–7384 (2013). 23736854 10.3390/s130607345 PMC3715274

[b30] Treeby B. E. & Cox B. T. k-Wave: MATLAB toolbox for the simulation and reconstruction of photoacoustic wave fields. Journal of biomedical optics 15, doi: Artn 02131410.1117/1.3360308 (2010). 10.1117/1.3360308 20459236

[b31] Ntziachristos V. & Razansky D. Molecular imaging by means of multispectral optoacoustic tomography (MSOT). Chemical reviews 110, 2783–2794 (2010). 20387910 10.1021/cr9002566

[b32] Bin J. *et al.* A laser-driven nanosecond proton source for radiobiological studies. Appl Phys Lett 101 (2012).

[b33] Bin J. H. *et al.* Ion acceleration using relativistic pulse shaping in near-critical-density plasmas. Phys Rev Lett 115, 4801–4801 (2015). 10.1103/PhysRevLett.115.064801 26296119

[b34] Omar M., Schwarz M., Soliman D., Symvoulidis P. & Ntziachristos V. Pushing the optical imaging limits of cancer with multi-frequency-band raster-scan optoacoustic mesoscopy (RSOM). Neoplasia 17, 208–214, doi: 10.1016/j.neo.2014.12.010 (2015). 25748240 PMC4351295

[b35] Heijblom M. *et al.* Photoacoustic image patterns of breast carcinoma and comparisons with Magnetic Resonance Imaging and vascular stained histopathology. Sci Rep 5, 11778, doi: 10.1038/srep11778 (2015). 26159440 PMC4498178

[b36] Reinhardt S., Hillbrand M., Wilkens J. J. & Assmann W. Comparison of Gafchromic EBT2 and EBT3 films for clinical photon and proton beams. Medical physics 39, 5257–5262 (2012). 22894450 10.1118/1.4737890

[b37] Herzog E. *et al.* Optical imaging of cancer heterogeneity with multispectral optoacoustic tomography. Radiology 263, 461–468 (2012). 22517960 10.1148/radiol.11111646

[b38] Wang L. V. Tutorial on photoacoustic microscopy and computed tomography. Ieee J Sel Top Quant 14, 171–179, doi: 10.1109/Jstqe.2007.913398 (2008).

[b39] Andreo P., Wulff J., Burns D. T. & Palmans H. Consistency in reference radiotherapy dosimetry: resolution of an apparent conundrum when Co-60 is the reference quality for charged-particle and photon beams. Phys Med Biol 58, 6593–6621 (2013). 24018471 10.1088/0031-9155/58/19/6593

