# Supplementary material for: Ionoacoustic tomography of the proton Bragg peak in combination with ultrasound and optoacoustic imaging
Source: Sci Rep. 2016 Jul 7;6:29305. doi: 10.1038/srep29305 (PMC4935843; doi:10.1038/srep29305)
Supplement: Supplementary Information [file srep29305-s3.pdf]

Ionoacoustic tomography of the proton Bragg peak in combination with ultrasound and optoacoustic imaging

Stephan Kellnberger[1,2†], Walter Assmann[3†], Sebastian Lehrack[3], Sabine Reinhardt[3], Peter Thirolf[3], Daniel Queirós[1], George Sergiadis[4], Günther Dollinger[5], Katia Parodi[3], Vasilis Ntziachristos[1*]

Supplementary Information

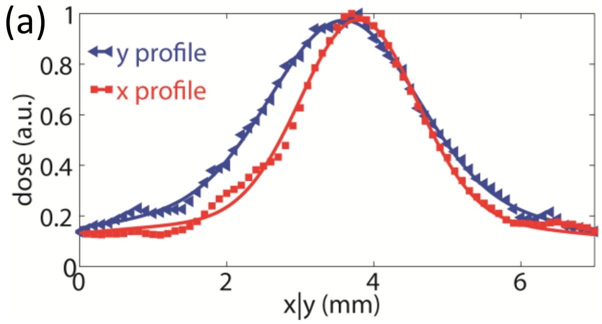

Figure S1. Line profiles of the Bragg peak from the ionoacoustic tomography experiment shown in Fig. 2. (**a**) Line profile of the Bragg peak in *x*- and *y*-direction from Fig. 2(b). The measurement points and Gaussian fits (triangle and squares) illustrate the full width half maximum (FWHM). In *x*-direction, we calculated the FWHM to be 2.8 ± 0.3 mm (red line) and in *y*-direction 2.0 ± 0.3 mm (blue line). (**b**) Line profile from Fig. 2(d). The FWHM in *x*-direction is 6.2 ± 0.5 mm and 6.1 ± 0.5 mm in *y*-direction.

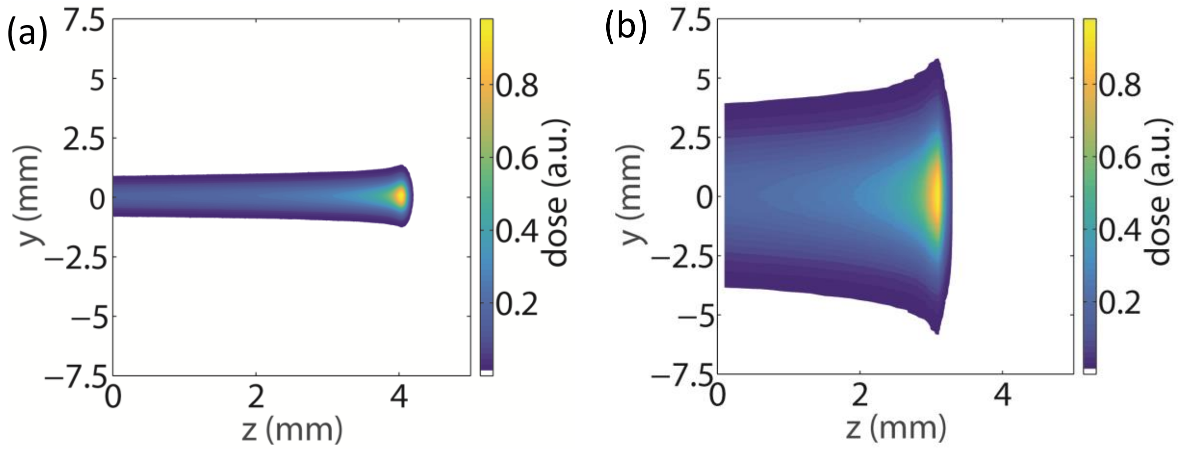

Figure S2. Geant4 simulations of the proton dose deposition in water. (**a**) 20 MeV dose deposition simulation. The range of protons after the entrance foil is dz = 4.06 mm. (**b**) 20 MeV dose deposition with Al sheet in beam axis. The corresponding range of protons is dz = 3.00 mm.

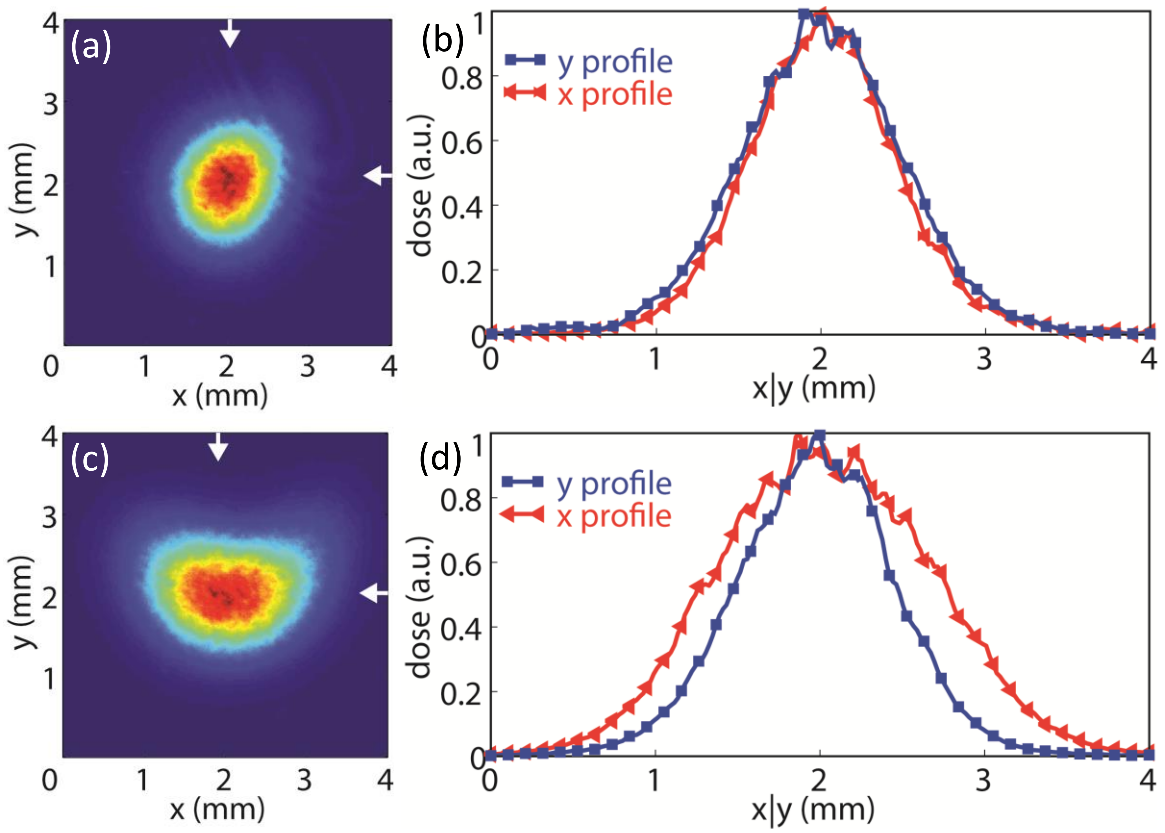

Figure S3. Radiochromic film measurements. (**a**) Film corresponding to ionoacoustic measurement shown in Fig. 1(b). (**b**) FWHM in *x*-direction as determined to 1.0 ± 0.2 mm and *y*-direction to 1.2 ± 0.2 mm. (**c**) Film corresponding to ionoacoustic tomographic experiment illustrated in Fig. 2(b). (**d**) The FWHM in *x*-direction was determined to 1.5 ± 0.2 mm and *y*-direction to 1.0 ± 0.2 mm.

Movie S1: 3D reconstruction of the Bragg peak employing the 64 element curved ultrasound array. The movie shows 36 scanning positions of the US array moved across the Bragg peak in steps sizes of 200 µm.

Movie S2: 3D reconstruction of the Bragg peak after introducing a 0.5 mm thick aluminum absorber into the beam. The movie shows 36 scanning steps of the US array scanned across the Bragg peak.
